# Lymphocyte-Depleted Hodgkin’s Lymphoma in Coeliac Disease: A Diagnostic Challenge During the COVID-19 Pandemic

**DOI:** 10.7759/cureus.28432

**Published:** 2022-08-26

**Authors:** Ana Cristina Mendes, Regina Costa, Ana Rita Ramalho, Nelson Pedro, Maria José Julião, Lèlita Santos

**Affiliations:** 1 Serviço de Medicina Interna, Centro Hospitalar e Universitário de Coimbra, Coimbra, PRT; 2 Serviço de Patologia Clínica, Centro Hospitalar e Universitário de Coimbra, Coimbra, PRT

**Keywords:** covid-19 pandemic, necrolytic migratory erythema, coeliac disease, lymphocyte-depleted hodgkin’s lymphoma, hodgkin’s lymphoma

## Abstract

A 51-year-old woman presented with constitutional symptoms, polydipsia, early satiety, nausea, vomiting, and a pruritic vesicular rash. On physical examination, she was febrile, had low peripheral oxygen saturation in room air (91%), hepatomegaly, lower limb edema, and palpable cervical adenopathies. She was hospitalized for diagnostic investigations and treatment. An autoimmune panel was requested which was positive for anti-parietal gastric cell, anti-endomysial, and anti-tissue transglutaminase antibodies, raising the suspicion for coeliac disease (CD). Gastric and duodenal biopsies were not diagnostic for CD, which was compatible with necrolytic migratory erythema similar to the vesicular rash biopsy. Thoracic-abdomino-pelvic computed tomography scan and fludeoxyglucose F18-positron emission tomography identified supra- and infra-diaphragmatic hypermetabolic adenopathies, with hypermetabolic activity in the lungs, suggestive of pulmonary lymphomatous involvement. Fine-needle aspiration of one supraclavicular adenopathy was performed but was not enough for histological diagnosis. The patient’s respiratory insufficiency worsened and she died on day 63 of hospitalization. The final diagnosis was achieved on an anatomopathological autopsy that showed lymphocyte-depleted Hodgkin’s lymphoma.

The association of CD with other lymphomas besides enteropathy-type T-cell lymphoma is not clear. There is no clear relationship between CD and lymphocyte-depleted Hodgkin’s lymphoma, which is the rarest subtype of classic Hodgkin’s lymphoma and, by itself, has a very poor prognosis. This case highlights the challenge in diagnosis and significant delay due to isolation associated with coronavirus disease 2019 infection.

## Introduction

Coeliac disease (CD) is an immune-mediated inflammatory condition of the small intestine caused by sensitivity to dietary gluten and related proteins in genetically predisposed individuals. Gluten-sensitive villous atrophy has been linked to a characteristic lymphoma of the small intestine referred to as enteropathy-type T-cell lymphoma (ETTL) [1]. Whether CD is associated with an increased risk of development of other lymphoma types is not clear [1]. In fact, to our knowledge, there is no described direct association between CD and lymphocyte-depleted Hodgkin’s lymphoma.

## Case presentation

**Figure 1:**
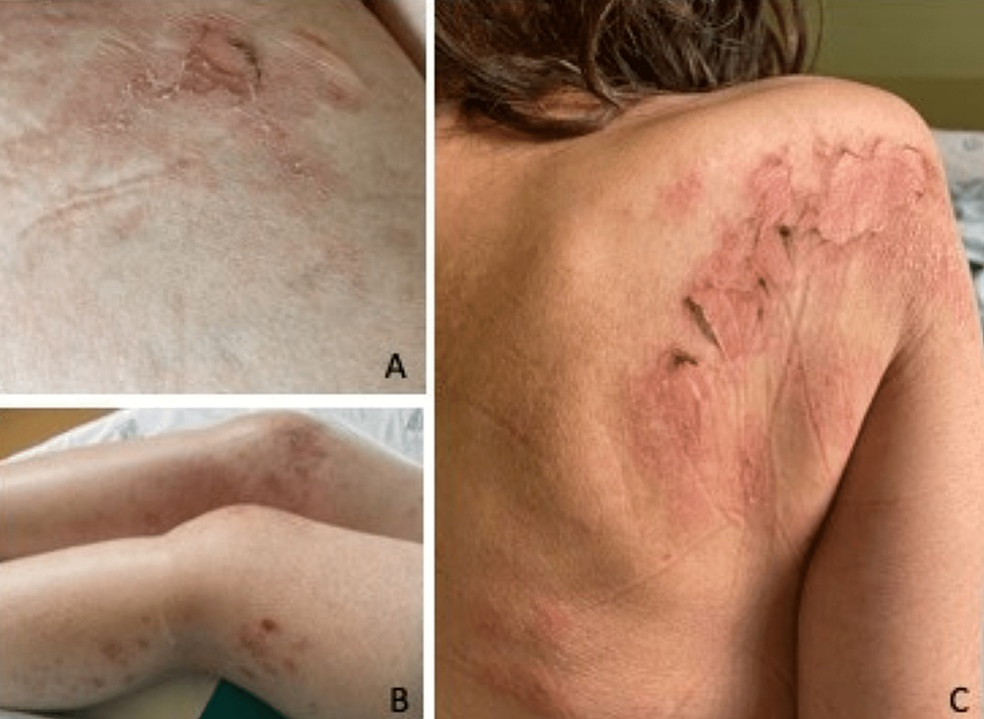
Necrolytic migratory erythema. A: Close-up of the inner thigh. B: Lower limbs bilaterally. C: Upper back.

A 51-year-old woman with a history of immune thrombocytopenic purpura, vitamin B12 deficiency anemia, and lactose intolerance presented to the emergency department with three weeks of constitutional symptoms such as loss of 10% of body weight over one month, polydipsia, early satiety, nausea, and vomiting. She had a pruritic vesicular rash in the skin folds and pressure areas. She denied having any other symptoms (Figure 1).

The patient was febrile (38.6°C), hypotensive (blood pressure 90/57 mmHg), and had low peripheral oxygen saturation in room air (91%). She appeared dehydrated and pale, with xerosis, hepatomegaly, lower limbs edema, and palpable cervical adenopathy. Initial blood analysis showed elevated inflammatory markers with discrete lymphopenia (Table 1).

**Table 1:** Results from the patient’s blood work during hospitalization.

Results	Day 1	Day 7	Day 9	Day 37	Day 58
Creatinine (mg/dL)	0.57	0.53	0.51	0.53	0.62
Sodium (mmol/L)	136	136	133	131	137
Potassium (mmol/L)	4.8	3.8	4.0	4.8	5.4
Lactate dehydrogenase (U/L)	386	540	639	443	571
Total protein (g/dL)	-	5.6	5.1	-	4.8
Albumin (g/dL)	-	2,6	2,3	-	2,5
Alkaline phosphatase (U/L)	269	246	393	188	1,246
Gamma-glutamyl transpeptidase (U/L)	-	41	79	48	181
Aspartate transaminase (U/L)	-	165	262	32	87
Alanine transaminase (U/L)	-	81	103	27	70
Total bilirubin (mg/dL)	0.5	0.5	0.6	0.5	2.1
C-reactive protein (mg/dL)	5.64	7.73	7.85	18.23	23.56
Procalcitonin (ng/mL)	-	-	1.75	-	
Leucocytes (×10^9^/L)	8.9	6.3	8.9	9.1	9.3
Lymphocytes (×10^9^/L)	0.83	0.76	-	-	
Hemoglobin (g/dL)		12.0	11.5	10.2	11.0
Ferritin (ng/mL)		1,808			
Folic acid (ng/mL)		1.8			
Vitamin B12 (pg/mL)		276			

**Figure 2:**
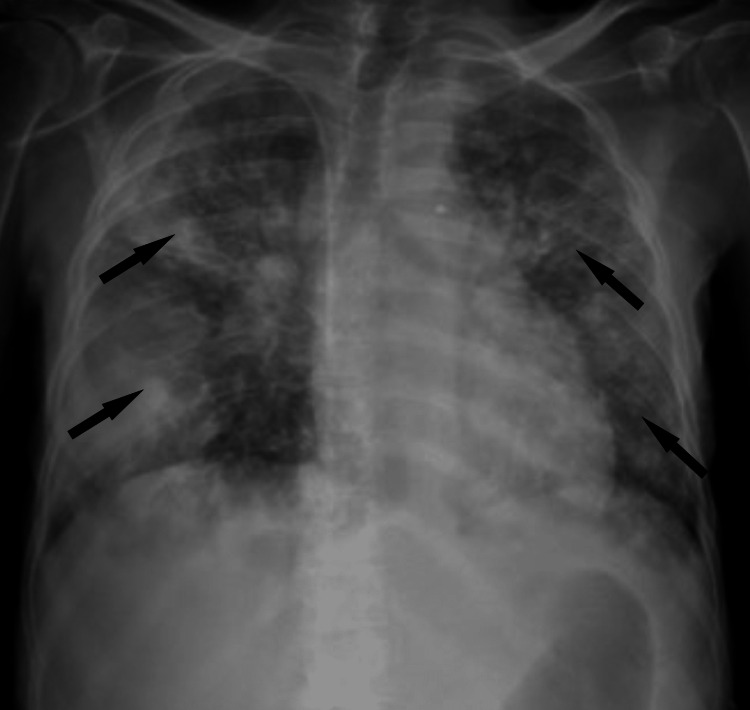
Chest X-ray. Apical to caudal interstitial infiltrates bilaterally (arrows).

Arterial blood gas showed hypoxemic respiratory failure and chest X-ray showed bilateral interstitial infiltrates, as shown in Figure 2.

Empiric antibiotic therapy with amoxicillin/clavulanate 1,200 mg 8/8 hours and azithromycin 500 mg 24/24 hours was initiated and the patient was hospitalized for diagnostic investigation and treatment.

On the fifth day of hospitalization, the patient’s respiratory condition worsened, which necessitated non-invasive ventilation (NIV). A chest computed tomography (CT) was performed which revealed multiple lymphadenopathies, consolidation with air bronchogram and ground-glass opacities, and thin pleural effusion on the right side. Severe acute respiratory syndrome coronavirus 2 (SARS-CoV-2) infection was detected in a nasal swab, and the patient was transferred to a COVID-19 care unit, which delayed the workup diagnosis for 20 days.

Infectious serologies for tuberculosis, human immunodeficiency virus, hepatitis B and C, *Borrelia burgdorferi*, *Brucella*, *Rickettsia*, *Bartonella*, *Coxiella*, Leishmaniasis, *Listeria monocytogenes*, *Mycoplasma pneumoniae*, *Legionella pneumophila*, Epstein-Barr virus, and cytomegalovirus were negative. Blood cultures were also negative. An autoimmune screen was also requested and was positive for anti-parietal gastric cells, anti-endomysial (EMA-IgA), and anti-tissue transglutaminase (tTG) antibodies. Search for HLA-DQ2.5 e HLA-DQ2.2 was homozygotic for DQB1*02, raising the high clinical suspicion of CD. Gastric and duodenum biopsies were performed to confirm the diagnosis and were both negative. Nevertheless, a gluten-free diet was started and the patient’s gastrointestinal symptoms improved. Skin biopsy of the vesicular rash identified necrolytic migratory erythema lesions associated with CD.

**Figure 3:**
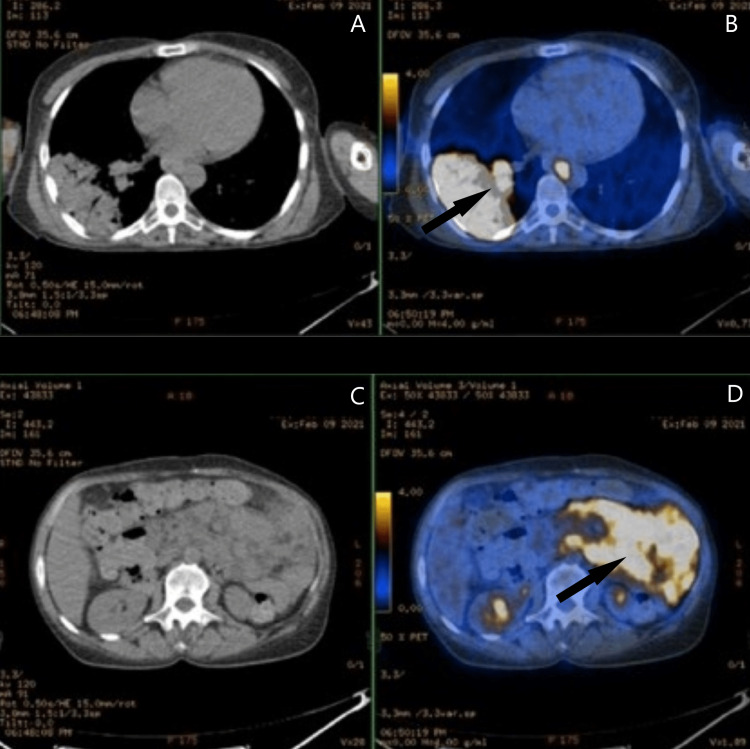
CT scan and FDG18-PET. A, B: Chest level showing right lower lobe lymphomatous infiltration (arrow). C, D: Abdominal level showing retroperitoneal adenopathy conglomerate (arrow). FDG18-PET = fludeoxyglucose F18-positron emission tomography

CT of the chest, abdomen, and pelvis showed an adenopathic conglomerate surrounding the mesenteric vessels, suggesting a diagnosis of lymphoproliferative disorder. This diagnosis was endorsed by fludeoxyglucose F18-positron emission tomography (FDG18-PET), which showed multiple supra- and infra-diaphragmatic hypermetabolic adenopathies and suggested pulmonary lymphomatous involvement (Figure 3).

Fine-needle aspiration of one of the supraclavicular lymph nodes was performed showing suggestive but not diagnostic findings of Hodgkin’s lymphoma.

The patient’s clinical condition worsened with the onset of diarrhea and worsening of lower limb edema. Only symptomatic treatment was offered due to the absence of a definitive diagnosis. The benefit of a lung biopsy was weighed, but the pulmonary condition and multiple respiratory complications contraindicated the procedure. The abdominal adenopathic conglomerate was inaccessible unless laparoscopic surgery was performed.

**Figure 4:**
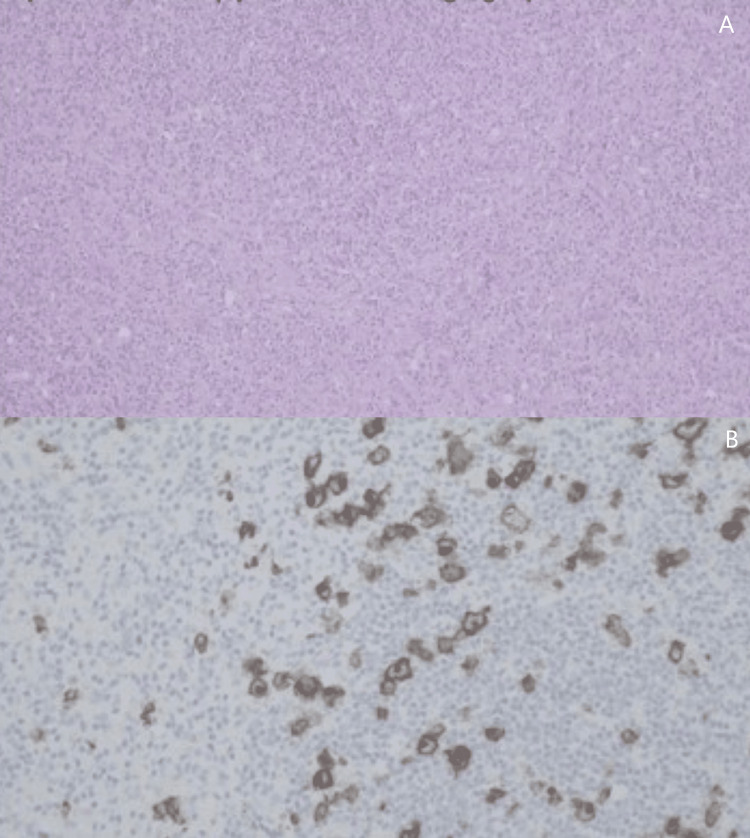
Anatomopathological autopsy. A: Hematoxylin and eosin stain of a cervical lymph node. B: CD30 differential stain; reticular fibrosis with areas of granulomatous appearance and scattered histiocytic cells.

Without any improvement in her clinical condition complicated by respiratory insufficiency, the patient died on day 63 of hospitalization. The final diagnosis was achieved on an anatomopathological autopsy that showed lymphocyte-depleted Hodgkin’s lymphoma, forming an adenopathic conglomerate in the retroperitoneum and abdomen with extensive involvement of the wall of the small intestine and the lung bilaterally. It also identified extensive involvement of the lung by lymphoma, with resulting respiratory failure which was the direct cause of death (Figure 4).

## Discussion

Lymphocyte-depleted Hodgkin’s lymphoma is the rarest subtype of classic Hodgkin’s lymphoma [2]. The majority of these patients are males over 60 years old and are in an advanced stage of the disease by the time the diagnosis is made. The diagnosis is challenging and the disease might be misdiagnosed as more aggressive B-cell or T-cell lymphomas [2]. Our case report highlights the challenging diagnosis. The patient was in respiratory isolation due to SARS-CoV-2 infection which resulted in an inability to obtain an adequate sample for histologic diagnosis. None of the tissue samples collected described either the Reed-Sternberg cells or the minimal lymphocyte infiltration and abundant histiocytes in a hypocellular background necessary for a definitive diagnosis of lymphocyte-depleted Hodgkin’s lymphoma [2].

Besides, in our case report, the diagnosis of CD was made through high clinical suspicion due to the history of vitamin B12 deficiency anemia, positive autoimmunity, and improvement of the gastrointestinal symptoms with a gluten-free diet, despite the negative gastric and duodenum biopsies [3]. Our patient’s necrolytic migratory erythema at the time of hospital admission is often associated with glucagonoma, but can also be seen in liver disease and intestinal malabsorption disorders [4].

Furthermore, pulmonary lymphomatous involvement is suggestive of lymphoid interstitial pneumonia (LIP), which is an extremely rare condition and easily misdiagnosed with primary malignant lymphoma of the lung, nonspecific interstitial pneumonia, pulmonary lymphangioleiomyomatosis, and pulmonary Langerhans cell histiocytosis. Primary LIP is rare, with most cases being secondary and usually associated with autoimmune diseases and viral infections. The mortality rate has been reported to be 33-50% within five years of diagnosis, and the transformation for malignant B-cell lymphoma is estimated to occur in 5% of the patients [5].

## Conclusions

Lymphomas complicating CD can be of any type and not only ETTL, requiring high clinical suspicion for the diagnosis in patients with CD. Moreover, the non-specific clinical and radiological features of LIP make it a challenging diagnosis, namely, in patients with interstitial infiltrates attributed to COVID-19 pneumonia. Moreover, this is a severe condition that may ultimately lead to the death of the patient due to respiratory failure, as shown in an anatomopathological autopsy.

Furthermore, our case reports an example of the delay in the diagnosis of some diseases during the COVID-19 pandemic, which is due not only to patients’ delay in the use of health services but also to the difficulty in accessing complementary diagnostic examinations for patients with SARS-CoV-2 infection.
